# *Radiation Oncology* reviewer acknowledgement 2013

**DOI:** 10.1186/1748-717X-9-53

**Published:** 2014-04-03

**Authors:** Claus Belka

**Affiliations:** 1 Munich, Germany

## Abstract

**Contributing reviewers:**

The editors of *Radiation Oncology* would like to thank all our reviewers who have contributed to the journal in Volume 8 (2013).

## 

Mehmet Ufuk Abacioglu

Turkey

Rimpa Achari

India

Daniel Aebersold

Switzerland

Sung Hwan Ahn

Korea South

Edwin Aird

United Kingdom

Francesca Albertini

Switzerland

Valerie Albrecht

Germany

Manuel Algara

Spain

Bilal Al-Nawas

Germany

Ravi Amaravadi

United States of America

Maurizio Amichetti

Italy

Richard Amos

United States of America

Fundagul Andic

Turkey

Nicolaus Andratschke

Germany

Wilhelm Ansorge

Switzerland

Hidefumi Aoyama

Japan

Carmen Ares

Switzerland

Cynthia Aristei

Italy

Molykutty Aryankalayil

United States of America

Sabrina Astner

Germany

Albert Attia

United States of America

Michele Avanzo

Italy

Batia Avni

Israel

David Azria

France

Eishi Baba

Japan

Emilia Bagiella

United States of America

Amit Bahl

United Kingdom

Kurt Baier

Germany

Elizabeth Baldini

United States of America

Mary Helen Barcellos-Hoff

United States of America

Guido Baroni

Italy

Alexandra Barratt

Australia

Rolf Barth

United States of America

Niels Bassler

Denmark

Yasser Bayoumi

Saudi Arabia

Stanley Benedict

United States of America

Mustafa Benekli

Turkey

Rene Jean Bensadoun

France

Tanya Berrang

Canada

Theocharis Berris

Austria

Christoph Bert

Germany

S¿Awomir Blamek

Poland

Oliver Blanck

Germany

Nils Bluthgen

Germany

Florian Bock

Austria

Judit Boda-Heggemann

Germany

Edwin Boelke

Germany

Till Tobias Böhlen

Austria

Françoise Bontemps

Belgium

Yerko Borghero

Christmas Island

Alberto Bossi

France

Dirk Bottke

Germany

Frank Boudghene

France

Barton Branstetter

United States of America

Priscilla Brastianos

United States of America

Klaus Bratengeier

Germany

Michael Bremer

Germany

Carsten Brink

Denmark

Martin Brown

United States of America

Krzysztof Bujko

Poland

Omer Burnett

United States of America

Edward Butler

United States of America

Elisabetta Cagni

Italy

Felipe Calvo

Spain

Raffaella Cambria

Italy

Antony Carver

United Kingdom

Jean-Michel Caudrelier

Canada

Crister Ceberg

Sweden

Laura Cella

Italy

Mustafa Cengiz

Turkey

Xiangfei Chai

United States of America

Anthony Chalmers

United Kingdom

Marc Chamberlain

United States of America

Maria Chan

United States of America

Albert Chang

United States of America

Joe Chang

United States of America

Zheng Chang

United States of America

Samuel Chao

United States of America

Naved Chaudhri

Germany

Fuxue Chen

China

Josephine Chen

United States of America

Ronald Chen

United States of America

Yuhchyau Chen

United States of America

Yu-Jen Chen

Taiwan

Stephen Chiang

United States of America

Eui Kyu Chie

Korea South

Nathan Childress

United States of America

Ronny Chin

United States of America

Youngmin Choi

Korea South

James Chow

Canada

Hans Christiansen

Germany

Caroline Chung

Canada

Heeteak Chung

United States of America

Peter Chung

Canada

Patrizia Ciammella

Italy

Savino Cilla

Italy

Alessandro Clivio

Switzerland

Brian Collins

United States of America

Sean Collins

United States of America

Stephanie E. Combs

Germany

Ulku Comelekoglu

Turkey

Gary Cook

United Kingdom

June Corry

Australia

Renzo Corvo

Italy

Cesare Cozzarini

Italy

Luca Cozzi

Switzerland

Gilles Crehange

France

Xing Cui

Japan

Juliane Daartz

United States of America

Giuseppe D'agostino

Italy

Alan Dal Pra

Switzerland

Roger Dale

United Kingdom

Rolando M. D'angelillo

Italy

Alexandru Dasu

Sweden

Gert De Meerleer

Belgium

Claudia Vitoria De Moura Gallo

Brazil

Maria De Ridder

Netherlands

Johannes De Wilt

Netherlands

Pradip Deb

Australia

Juergen Debus

Germany

Charles Deehan

United Kingdom

Robert Den

United States of America

J Keith Dewyngaert

United States of America

Mantana Dhanachai

Thailand

Fernanda Dias Gonzalez Guindalini

United States of America

Adam Dicker

United States of America

Eric Diffenderfer

United States of America

Johannes Carl Athanasios Dimopoulos

Greece

Kai Ding

United States of America

Francesco Dionisi

Italy

Kevin Du

United States of America

Jean-Bernard Dubois

France

Marciana Nona Duma

Germany

Peter Dunscombe

Canada

Fréderic Duprez

Belgium

Marco Durante

Germany

Marie Dutreix

France

Oleksandr Dzyubak

United States of America

Janet Eary

United States of America

Franziska Eckert

Germany

Karsten Eilertsen

Norway

Avraham Eisbruch

United States of America

Iris Eke

Germany

John Eley

United States of America

Benedikt Engels

Belgium

Rita Engenhart-Cabillic

Germany

Ralph Ermoian

United States of America

Floris Ernst

Germany

Frank Essmann

Germany

François Estève

France

Prasad Eswaran

India

Philip Evans

United Kingdom

Muthuveni Ezhil

United Kingdom

Carlo Fallai

Italy

Sergio Faria

Canada

Dimitrios Fatouros

Greece

Malcolm Feigen

Australia

Pascal Fenoglietto

France

Gerardo Ferbeyre

Canada

Vladimir Feygelman

United States of America

Rainer Fietkau

Germany

Andrea Riccardo Filippi

Italy

Claudio Fiorino

Italy

David Fitzpatrick

Ireland

Michael K Fix

Switzerland

Tom Flannery

United Kingdom

Jacob Flanz

United States of America

Jochen Fleckenstein

Germany

Shannon Fogh

United States of America

Valérie Fonteyne

Belgium

Nicolas Foray

France

Gregory Fortier

United States of America

Piero Fossati

Italy

Giovanni Franchin

Italy

Michael Freeman

United States of America

Benjamin Frey

Germany

Anna Friedl

Germany

Weihua Fu

United States of America

Xiao-Long Fu

China

Simone Fulda

Germany

Ingrid Fumagalli

France

Frank Gagliardi

Australia

Udo Gaipl

Germany

Razvan Galalae

Germany

Sergio Gallardo

Spain

Maria Antonietta Gambacorta

Italy

Robin Garcia

France

Tobias Gauer

Germany

Domenico Genovesi

Italy

Dietmar Georg

Austria

Petra Georg

Austria

Gargi Ghosal

United States of America

Sarbani Ghosh Laskar

India

David Gierga

United States of America

Jordi Giralt

Spain

Michael Girvigian

United States of America

Kristina Giske

Germany

Rosalind Glasspool

United Kingdom

Daniel Gomez

United States of America

Karyn Goodman

United States of America

Alexander Gottschalk

United States of America

Gerhard Grabenbauer

Germany

Christian Graeff

Germany

Joel Greenberger

United States of America

Steffen Greilich

Germany

Stephan Gripp

Germany

Christian Grommes

United States of America

David Grosshans

United States of America

Arne Gruen

Germany

Ferran Guedea

Spain

Tejpal Gupta

India

Cornelis Haasbeek

Netherlands

Daniel Habermehl

Germany

Janet Hall

France

Christopher Hallemeier

United States of America

Randala Hamdan

United States of America

Hassan Hamdy

Egypt

Chunhui Han

United States of America

Gerard G. Hanna

United Kingdom

Vibeke Hansen

United Kingdom

Hideyuki Harada

Japan

Nadia Harbeck

Germany

Nicholas Hardcastle

Australia

William Hartsell

United States of America

Joan Hatton

Australia

Henrik Hauswald

Germany

Uwe Haverkamp

Germany

Maria Hawkins

United Kingdom

Kazushige Hayakawa

Japan

Barbara Head

United States of America

Rajendra Hegde

Australia

Stephanie Hehlgans

Germany

Guido Henke

Germany

Jaroslaw Hepel

United States of America

Dwight Heron

United States of America

Daniel Higginson

United States of America

Elif Hindie

France

Naoki Hirasawa

Japan

Hideki Hirata

Japan

Ying Hitchcock

United States of America

Ivy Ho

Singapore

Ulrike Hoeller

Germany

Sarah Hoffe

United States of America

Sabine Hornhardt

Germany

Janet Horton

United States of America

Morten Høyer

Denmark

Chen-Hsi Hsieh

Taiwan

Ching-Yeh Hsiung

Taiwan

Fred Hsu

Canada

Chaosu Hu

China

Kitty Huang

Canada

Shenghai Huang

China

Yu-Jie Huang

Taiwan

Coen Hurkmans

Netherlands

Dominique Huyskens

Belgium

Ui-Jung Hwang

Korea South

Jan Ijzermans

Netherlands

Luca Incrocci

Netherlands

Daniel Indelicato

United States of America

Hiroshi Inoue

Japan

Edy Ippolito

Italy

Sofie Isebaert

Belgium

Hiromichi Ishiyama

Japan

Alesia Ivashkevich

Australia

Pooja Jain

United Kingdom

Suneil Jain

United Kingdom

Mahshid Jalilian

Australia

Reiner Jänicke

Germany

Nora Janjan

United States of America

Nathalie Jansen

Germany

Urszula Jelen

Germany

Verena Jendrossek

Germany

Alexandra D. Jensen

Germany

Hosang Jin

United States of America

Subhash John

India

Marjory Jolicoeur

Canada

Bernard Jones

United States of America

Robin Jones

United States of America

Christian Jorgensen

France

Shalom Kalnicki

United States of America

Tadashi Kamada

Japan

Wai Kwan Kan

Hong Kong

Dehua Kang

China

Young Nam Kang

Korea South

Christian P. Karger

Germany

Shingo Kato

Japan

Alan Katz

United States of America

Shinji Kawabata

Japan

Chul-Seung Kay

Korea South

Lucyna Kepka

Poland

Lucyna Kepka

Poland

Ki Chang Keum

Korea South

Chan Kyo Kim

Korea South

Kyubo Kim

Korea South

Mi-Sook Kim

Korea South

Siyong Kim

United States of America

Wontaek Kim

Korea South

Yoshihiro Kinoshita

Japan

Linda Kinzel

Germany

Christian Kirisits

Austria

Barbara Knäusl

Austria

Christin Knowlton

United States of America

Bridget Koontz

United States of America

Rolf-Dieter Kortmann

Germany

Gyoergy Kovacs

Germany

Gerhard Kraft

Germany

Mechthild Krause

Germany

Andra Krauze

United States of America

Robert Krempien

Germany

Marco Krengli

Italy

Tomas Kron

Australia

Kevin Krull

United States of America

Stephen Kry

United States of America

Sanath Kumar

United States of America

Meral Kurt

Turkey

Abraham Kuten

Israel

Yok Lam Kwong

China

Michael Lace

United States of America

Jan Lagendijk

Netherlands

Chyong-Huey Lai

Taiwan

Seeta Lakka

United States of America

Rachelle Lanciano

United States of America

Christopher Lange

United States of America

Britta Langen

Sweden

Hans Langendijk

Netherlands

George Laramore

United States of America

Maria Larsson

Sweden

Arnon Lavie

United States of America

Stephen Law

Hong Kong

Richard Yaacov Lawrence

United States of America

Wolfgang Lechner

Austria

Sang Yeob Lee

United States of America

Yi-Jang Lee

Taiwan

Andreas Leithner

Austria

Rolf Lewensohn

Sweden

David Lewis

United Kingdom

Allen Li

United States of America

Chuan-Yuan Li

United States of America

Guang-Hui Li

China

Harold Li

United States of America

Jun Li

United States of America

Li Li

China

Minglun Li

Germany

Taoran Li

United States of America

Jun Lian

United States of America

Xiaoying Liang

United States of America

Hendrik Lieshout

Netherlands

Eva Lieto

Italy

Qiang Lin

China

Katja Lindel

Germany

Mark Linskey

United States of America

Chihray Liu

United States of America

Chun-Feng Liu

China

Guangwei Liu

China

Qing-Jie Liu

China

Lorenzo Livi

Italy

Simon Lo

United States of America

Ashildur Logadottir

Denmark

Frank Lohr

Germany

Max Lonneux

Belgium

Maria Do Carmo Lopes

Portugal

Jose Luis Lopez Guerra

Spain

Sandra Losa

France

Bo Lu

United States of America

Hsiao-Ming Lu

United States of America

Jiade Lu

Singapore

Ld Lunsford

United States of America

L. Dade Lunsford Md

United States of America

Jinli Ma

China

Jun Ma

China

Cornelius Maihöfer

Germany

Philippe Maingon

France

Vicky Makker

United States of America

Gregoire Malandain

France

Julian Malicki

Poland

Eirik Malinen

Norway

Pietro Mancosu

Italy

Kailash Manda

India

Stephen Mangar

United Kingdom

Karen Marcus

United States of America

Carl Marfurt

United States of America

Simone Marnitz

Germany

Maria Martisikova

Germany

Dai Maruyama

Japan

Valerie Matarese

Italy

Yukinori Matsuo

Japan

Peter Matt

Switzerland

Joan Maurel

Spain

Alejandro Mazal

France

Jonathan Mcaleese

United Kingdom

William Mcbride

United States of America

Philip Mccloud

Australia

Stephen Mcmahon

United Kingdom

Tamene Melkamu

United States of America

Cynthia Menard

Canada

Susanne Merkel

Germany

Juergen Meyer

United States of America

Raymond Meyn

United States of America

Aisha Miah

United Kingdom

Oliver Micke

Germany

Petra Miglierini

France

Tom Mikkelsen

United States of America

Michael Milano

United States of America

Dusan Milanovic

Germany

Robert C Miller

United States of America

Tatsuya Mimura

United States of America

Giuseppe Minniti

Italy

Raymond Miralbell

Switzerland

Junetsu Mizoe

Japan

Raphaël Moeckli

Switzerland

Mohammad Mohammadianpanah

Iran

David Mönnich

Germany

Alessio G. Morganti

Italy

Shinichiro Mori

Japan

Gerard Morton

Canada

Mukesh Mukesh

United Kingdom

Arndt-Christian Müller

Germany

Gabriele Multhoff

Germany

Per Munck Af Rosenschöld

Denmark

Martin Murphy

United States of America

Olaf Nairz

Austria

Keiichi Nakagawa

Japan

Katsumasa Nakamura

Japan

Mitsuhiro Nakamura

Japan

Naoki Nakamura

Japan

Joo-Hyun Nam

Korea South

Chiara Napoletano

Italy

Kailash Narayan

Australia

Alfons Navarro

Spain

Alexander Nevelsky

Israel

Nam Nguyen

United States of America

R. Charles Nichols

United States of America

Carsten Nieder

Norway

Peter Niehoff

Germany

Marcus Niewald

Germany

Simeon Nill

United Kingdom

Ajay Niranjan

United States of America

Maximilian Niyazi

Germany

Angelica Nogueira-Rodrigues

Brazil

Takuma Nomiya

Japan

Sandra Nuyts

Belgium

Joost Nuyttens

Netherlands

Tufve Nyholm

Sweden

Richard Oates

Australia

Etsuyo Ogo

Japan

Robert Olson

Canada

Hiroshi Onishi

Japan

Ester Orlandi

Italy

Michael Orth

Germany

Pedro Ortiz Lopez

Austria

Feras Oskan

Germany

Piet Ost

Belgium

Mattia Falchetto Osti

Italy

Joe O'sullivan

United Kingdom

Mahmut Ozsahin

Switzerland

Enis Ozyar

Turkey

Gokhan Ozyigit

Turkey

Jianji Pan

China

Badri Pandey

India

Valerie Panet-Raymond

Canada

Nikos Papanikolaou

United States of America

Frank Papatheofanis

United States of America

David Pasquier

France

Samir Patel

Canada

Shilpen Patel

United States of America

Frank Paulsen

Germany

Piernicola Pedicini

Italy

Berrin Pehlivan

Turkey

Cassio Pellizzon

Brazil

Jose Penagaricano

United States of America

Yahui Peng

United States of America

Vincent Pennaneach

France

Richard Penson

United States of America

Marta Peroni

Italy

Lucas Persoon

Netherlands

Nadeem Pervez

Canada

Cordula Petersen

Germany

Jørgen Petersen

Denmark

Heike Peulen

Netherlands

Raphael Pfeffer

Israel

Justin Phillips

United States of America

Alessia Pica

Switzerland

Jean-Philippe Pignol

Canada

Emanuele Pignoli

Italy

Steffi Ulrike Pigorsch

Germany

Michael Pinkawa

Germany

Matthias Pinter

Austria

Fabian Pitoia

Argentina

Ludwig Plasswilm

Switzerland

George Plataniotis

United Kingdom

Buelent Polat

Germany

Bjoern Poppe

Germany

Paola Porcari

Italy

Louis Potters

United States of America

Christoph Pöttgen

Germany

Per Rugaard Poulsen

Denmark

Vesna Prokic

Germany

Claudio Pusceddu

Italy

Paul Martin Putora

Switzerland

Peng Qi

United States of America

Chao-Nan Qian

United States of America

Cornelis Raaijmakers

Netherlands

Dirk Rades

Germany

Roy Rampling

United Kingdom

Tiziana Rancati

Italy

Coen Rasch

Netherlands

Paul Read

United States of America

Christophe Redon

United States of America

Anne Hansen Ree

Norway

William Regine

United States of America

Roland Reitsamer

Austria

Martin Reuter

United States of America

David Riddle

United States of America

Kent Riley

United States of America

Mark Rivard

United States of America

Mack Roach

United States of America

James Robar

Canada

Nathalie Rochet

United States of America

Franz Rödel

Germany

Falk Roeder

Germany

Buck Rogers

United States of America

Yi Rong

United States of America

Florin Rosca

United States of America

Stephan Roth

Germany

Angeles Rovirosa

Spain

Domenico Rubello

Italy

Jeremy Ruben

Australia

Justine Rudner

Germany

Eva Rutkowska

United Kingdom

Eva Rutkowska

United Kingdom

Jean-Claude Rwigema

United States of America

Ramaswamy Sadagopan

United States of America

Haneen Sadick

Germany

Hideyuki Sakurai

Japan

Joao Salvajoli

Brazil

Henning Salz

Germany

John Sampson

United States of America

Riccardo Santoni

Italy

Jann Sarkaria

United States of America

Masataka Sawaki

Japan

Devin Schellenberg

Canada

Guenter Schreier

Austria

Reinhard Schulte

United States of America

Daniela Schulz-Ertner

Germany

Marco Schwarz

Italy

Amanda Schwint

Argentina

Marta Scorsetti

Italy

Felix Sedlmayer

Austria

Therese Seierstad

Norway

Ugur Selek

Turkey

Sabine Semrau

Germany

Suresh Senan

Netherlands

Sashendra Senthi

Australia

Natalie Serkova

United States of America

Masoud Shariat

Malaysia

Vinay Sharma

South Africa

Michael Sharpe

Canada

Zhiyuan Shen

United States of America

Yuta Shibamoto

Japan

Helen Shih

United States of America

Naoya Shikazono

Japan

Eric Shinohara

United States of America

Susan Short

United Kingdom

Mark Sidhom

Australia

Marco Andrea Signor

Italy

Anna Simeonova

Germany

Ben Slotman

Netherlands

Wendy Smith

Canada

Matthias Soehn

Germany

Davendra Sohal

United States of America

Thomas Sokollik

China

Shiyu Song

United States of America

Yongchun Song

China

Aaron Spalding

United States of America

Lena Specht

Denmark

Daniel Spratt

United States of America

Gabriele Sroka-Perez

Germany

Johannes Stahl

United States of America

Robert Staton

United States of America

Elisabeth Steiner

Austria

Kevin Stephans

United States of America

Florian Sterzing

Germany

Florian Stieler

Germany

Nelson Stone

United States of America

Guy Storme

Belgium

Lidia Strigari

Italy

Vratislav Strnad

Germany

Gabriela Studer

Switzerland

Martin Stuschke

Germany

Shanmuga Subramanian

India

Dacita Suen

Hong Kong

Marta Switlyk

Norway

Sheng-Hong Tseng

Taiwan

Phillip Taddei

Lebanon

Fumitaka Takeshita

Japan

Litee Tan

United Kingdom

Kari Tanderup

United States of America

Shikui Tang

United States of America

Marco Tatullo

Italy

Daniel Taussky

Canada

Roger Taylor

United Kingdom

Bin S. Teh

United States of America

Teruki Teshima

Japan

Ivan Tham

Singapore

Fabian J Theis

Germany

Sara Thoernqvist

Norway

Geraldine Thomas

United Kingdom

Wade Thorstad

United States of America

Uwe Titt

United States of America

Kensei Tobinai

Japan

Zelig Tochner

United States of America

Takafumi Toita

Japan

Koichi Tokuuye

Japan

Iuliana Toma-Dasu

Sweden

Vincenzo Tombolini

Italy

Natsuo Tomita

Japan

Erkan Topkan

Turkey

Masamichi Toshima

Japan

David Tougeron

France

Mahmoud Toulany

Germany

Edouard Trabulsi

United States of America

Alison Tree

United Kingdom

Dragan Trivanovic

Croatia

Nikolaos Tselis

Germany

Pelagia Tsoutsou

Switzerland

Audrey Tsunoda

Brazil

Jeffrey Tuan

Italy

Yee Ung

Canada

Kristian Unger

Germany

Diclehan Unsal

Turkey

Stefano Ursino

Italy

Julien Uzan

United Kingdom

Antti Vaheri

Finland

Uulke Van Der Heide

Netherlands

Hans Paul Van Der Laan

Netherlands

Wouter Van Elmpt

Netherlands

Dirk Van Gestel

Belgium

Birgitt Van Oorschot

Germany

Charles Van Praet

Belgium

Peter Vavassis

Canada

Vaneja Velenik

Slovenia

Dirk Verellen

Belgium

Kristina Viktorsson

Sweden

Francois Vincent

Canada

Vincent Vinh-Hung

Martinique

Akila Viswanathan

United States of America

Dirk Vordermark

Germany

Huan Vu

United States of America

Hsin-Ell Wang

Taiwan

Jufang Wang

China

Ya Wang

United States of America

Samantha Warren

United Kingdom

Colin Watts

United Kingdom

Damien Charles Weber

Switzerland

Joseph Wee

Singapore

Thomas G. Wendt

Germany

Maria Werner-Wasik

United States of America

David Wertheim

United Kingdom

Lukasz Wieczorkowski

Germany

Ruud Wiggenraad

Netherlands

Henrik Wilms

United States of America

Paul Wilson

United States of America

Olaf Witt

Germany

Philip Wong

Canada

Shiao Woo

United States of America

Jackson Wu

Canada

Ping Xia

United States of America

Feng Xu

China

Xiao-Ying Xue

China

Poonam Yadav

United States of America

Di Yan

United States of America

Guanghua Yan

United States of America

Gary Yang

United States of America

Ruijie Yang

China

Xinming Yang

China

Yong Yang

United States of America

Zhenzhou Yang

China

Fang-Fang Yin

United States of America

Lingshu Yin

United States of America

Yoshihito Yokoyama

Japan

Shunsuke Yonai

Japan

Sua Yoo

United States of America

Ken Yoshida

Japan

Cedric Yu

United States of America

Jinming Yu

China

Siu Ki Yu

Hong Kong

Yu Yuan

United States of America

Ning Yue

United States of America

Angelika Zabel-Du Bois

Germany

Nikolaos Zamboglou

Germany

Verena Zangen

Germany

Sergei Zavgorodni

Canada

Michael Zelefsky

United States of America

Cai Zhang

China

Haojian Zhang

United States of America

Hong Zhang

United States of America

Xiaodong Zhang

United States of America

Boris Zhivotovsky

Sweden

Yuan Zhiyong

China

Hong Zhou

China

Jinyuan Zhou

United States of America

Ping-Kun Zhou

China

Thomas Zilli

Switzerland

Frank Zimmermann

Switzerland

Faruk Zorlu

Turkey

Daniel Zwahlen

Switzerland

